# Effect of Spatial and Spectral Scaling on Joint Characterization of the Spectral Mixture Residual: Comparative Analysis of AVIRIS and WorldView-3 SWIR for Geologic Mapping in Anza-Borrego Desert State Park

**DOI:** 10.3390/s23156742

**Published:** 2023-07-28

**Authors:** Jeffrey Price, Daniel Sousa, Francis J. Sousa

**Affiliations:** 1 Department of Geography, San Diego State University, San Diego, CA 92182, USA; jcprice@sdsu.edu; 2 College of Earth, Ocean and Atmospheric Sciences, Oregon State University, Corvallis, OR 97331, USA; francis.sousa@oregonstate.edu

**Keywords:** imaging spectroscopy, hyperspectral, AVIRIS, WorldView-3, spectral mixture residual, joint characterization

## Abstract

A geologic map is both a visual depiction of the lithologies and structures occurring at the Earth’s surface and a representation of a conceptual model for the geologic history in a region. The work needed to capture such multifaced information in an accurate geologic map is time consuming. Remote sensing can complement traditional primary field observations, geochemistry, chronometry, and subsurface geophysical data in providing useful information to assist with the geologic mapping process. Two novel sources of remote sensing data are particularly relevant for geologic mapping applications: decameter-resolution imaging spectroscopy (spectroscopic imaging) and meter-resolution multispectral shortwave infrared (SWIR) imaging. Decameter spectroscopic imagery can capture important mineral absorptions but is frequently unable to spatially resolve important geologic features. Meter-resolution multispectral SWIR images are better able to resolve fine spatial features but offer reduced spectral information. Such disparate but complementary datasets can be challenging to integrate into the geologic mapping process. Here, we conduct a comparative analysis of spatial and spectral scaling for two such datasets: one Airborne Visible/Infrared Imaging Spectrometer—Classic (AVIRIS-classic) flightline, and one WorldView-3 (WV3) scene, for a geologically complex landscape in Anza-Borrego Desert State Park, California. To do so, we use a two-stage framework that synthesizes recent advances in the spectral mixture residual and joint characterization. The mixture residual uses the wavelength-explicit misfit of a linear spectral mixture model to capture low variance spectral signals. Joint characterization utilizes nonlinear dimensionality reduction (manifold learning) to visualize spectral feature space topology and identify clusters of statistically similar spectra. For this study area, the spectral mixture residual clearly reveals greater spectral dimensionality in AVIRIS than WorldView (99% of variance in 39 versus 5 residual dimensions). Additionally, joint characterization shows more complex spectral feature space topology for AVIRIS than WorldView, revealing information useful to the geologic mapping process in the form of mineralogical variability both within and among mapped geologic units. These results illustrate the potential of recent and planned imaging spectroscopy missions to complement high-resolution multispectral imagery—along with field and lab observations—in planning, collecting, and interpreting the results from geologic field work.

## 1. Introduction

Geologic maps visualize a diverse spectrum of Earth information, including bedrock, surficial mineralogy, and geologic structures like faults, folds, and fractures [1]. The information represented by geologic maps underlies the global economy, with applications at the core of diverse fields such as the entirety of our built environment (steel, concrete, glass, wallboard, transport, and all petrochemicals including fuels and downstream derivatives), water resources (hydrology, surface and ground water transport and quality), and critical metals’ extraction like those required for all modern electronics. Given these important uses, geologists are always seeking to improve upon mapping techniques and workflows to maximize the accuracy and precision of the conceptual models that geologic maps represent.

Remote sensing has been used for decades to inform geologic field campaigns with spatially extensive structural and lithological information [2]. In particular, the spatially continuous nature of airborne and satellite imaging can complement the model of discretizing map units as is required for drafting unit boundaries on a geologic map [2,3]. In the ideal scenario, remote sensing can best be used in conjunction with comprehensive field measurements to both inform pre-campaign planning and post-campaign analysis (e.g., [4]).

Since the launch of the first Landsat satellite in 1972, geologically relevant multispectral imagery has been available for those with the need—and the financial wherewithal, until the opening of the Landsat archive—to understand and characterize the Earth’s surface [5]. Seminal studies focusing on well-characterized areas like Cuprite, NV, have illustrated the ability of multispectral and spectroscopic imagery to capture specific absorption features and discriminate among rock types [6]. Spectral imaging has been used to detect lineaments [2] and describe mineralogy on Earth [6] and beyond [7].

Satellite imaging spectroscopy (IS) [8] is rapidly developing, with international missions like ISA’s PRISMA [9], ESA’s CHIME [10], DLR’s DESIS [11] and EnMAP [12], and JAXA’s HISUI [13] complemented by NASA missions like EMIT [14] and the planned Surface Biology and Geology (SBG) [15]. As a result, global high-quality satellite imaging spectroscopy (IS, or spectroscopic imaging) is rapidly becoming available. Optimal approaches for effective analysis of these data, including synthesis with multispectral imagery, will be of paramount importance moving forward.

A wide range of approaches exist for the use of IS data in geologic applications. Algorithms for identification and quantification of mineral abundance (e.g., Tetracorder) have shown decades of success in creating detailed mineralogical maps [16]. While offering a wealth of information, such algorithms largely operate through pixel-by-pixel matching of remotely sensed reflectance spectra to a spectral library of known minerals. Pixel-level analysis qualitatively differs from analysis at the scale of geologic map units, which can be highly variable in order to match the complex reality of the rocks on the ground. For those seeking to understand bedrock relations and geologic history, delineating discontinuities that distinguish between rock units in the field is often more useful than a measure of bulk geochemistry and mineralogy of the ground sample area represented by a pixel [3,4]. Decades of research have also resulted in a wide range of statistically-motivated mapping algorithms, ranging from statistical endmember selection approaches like the pixel purity index [17], to discrete image classifiers such as support vector machines [18] and neural networks trained to identify lithology [19]. Despite some improvement in the stated classification accuracy, such statistical algorithms are fundamentally data-driven and thus frequently less physically interpretable than optical models rooted in first principles. Ideally, physical and statistical approaches can be used together to maximize their utility for a diverse range of geologic mapping purposes.

Recent advances in IS data analysis may provide a synergistic path forward by combining the spectral mixture residual (MR) and joint characterization (JC). Briefly, the MR seeks to model the spectral continuum using a physically meaningful linear mixture model, and also retain the (low variance) wavelength-explicit vector residual of this model [20]. This is in contrast with traditional approaches to spectral mixture analysis which summarize model misfit as a single (typically root-mean-square) scalar metric. JC focuses on the high-dimensional topology (manifold structure) of the data, employing nonlinear dimensionality reduction to retain the (statistically) local connectivity structure of the data [21]. Recent work has shown that, for some mapping applications, JC and MR can be used together to provide mutually complementary geologic information, leading to enhanced clustering in the statistical domain and characterization of certain rock units and formations in the physical domain [4].

Despite these recent advances, important questions remain. In particular, the sensitivity of this approach to spatial and spectral resolution, and the potential complementarity of datasets with differing spatial and spectral resolutions for outcrop characterization, remain outstanding. Here, we seek to address these important questions using coincident decameter-scale imaging spectroscopy (AVIRIS-classic) and meter-scale multispectral SWIR (WorldView-3) datasets in a geologically complex setting in Anza-Borrego State Park, California. Specifically, we seek to answer the following questions:

1. To what extent does the greater spectral range (380–2500 nm) and finer spectral resolution (10 nm) of AVIRIS contain useful geologic information that is not present in 8-band multispectral SWIR data from WorldView-3? What spectral information is retained by both sensors? What does this tell us about the wavelength (visible through near-infrared (VNIR) vs. shortwave infrared (SWIR)) and bandwidth (narrow absorptions vs. continuum curvature) of geologically meaningful absorption features in this area?
2. How does variance-based characterization (Principal Component Analysis, PCA) differ from manifold-based characterization (Uniform Manifold Approximation and Projection, UMAP) for geologic mapping with both datasets? How does application of the spectral mixture residual clarify—or not—information in AVIRIS versus WorldView-3? What, if any, information emerges through joint characterization that could not be accessed through each technique by itself?
3. What new information is gained through the applications explored in this study that is useful to a geologist? Specifically, in what context might this methodology be useful for planning and executing a geologic mapping campaign?

The paper proceeds as follows. First, we discuss the data used for our analysis and their acquisition. Then, we outline the methodology for how we analyzed and processed the imagery, which was the same for spectroscopic and multispectral. This is supplemented by the results at each step, as well as a brief discussion and comparison of the results for each sensor. Finally, we contextualize the results in terms of their utility both to remote sensing specialists and geologists, as well as how our methodology could be useful in the upcoming wave of remote sensing technology and data.

## 2. Materials and Methods

### 2.1. Data 

#### 2.1.1. Image Data

Airborne imaging spectroscopy data were collected by the Airborne Visible Infrared Imaging Spectrometer (AVIRIS-classic) on 25 June 2018. Level-2 ATmospheric REMoval algorithm (ATREM)-corrected [22] surface reflectance was downloaded free of charge from the JPL data portal at: https://aviris.jpl.nasa.gov/dataportal/ (accessed on 1 July 2022). The flight azimuth was approximately NW to SE. The local time of collection was approximately 11:07 a.m., with solar elevation 75.75°. The ground sampling distance (GSD) was approximately 15.7 m. The flightline ID is provided in Appendix A.

WorldView-3 (WV3) multispectral shortwave infrared (SWIR) data were collected on 27 October 2018. Approximately 8 SWIR bands were collected, with band center wavelengths ranging from 1195 to 2365 nm. The nadir-looking GSD was approximately 3.7 m. Data were collected 24.7° off-nadir, resulting in a ground sampling distance of approximately 4.2 m. The solar elevation was 43°. WV3 data were obtained upon request to the NASA Commercial Smallsat Data Program (CSDA) at https://www.earthdata.nasa.gov/esds/csda (accessed on 1 July 2022). The scene ID and other metadata are provided in Appendix A.

#### 2.1.2. Geologic Maps and Vector Data

The published geologic maps were viewed and downloaded using the MapView interface provided by the National Geologic Map Database [23]. The geologic maps used were at 62,500 scale [24] and 250,000 scale [25]. The vector boundary of Anza-Borrego Desert State Park (AB) was obtained as a shapefile from the 2022 California Protected Areas Database (CPAD) [26].

## 3. Results

### 3.1. Preprocessing

**Figure 1:**
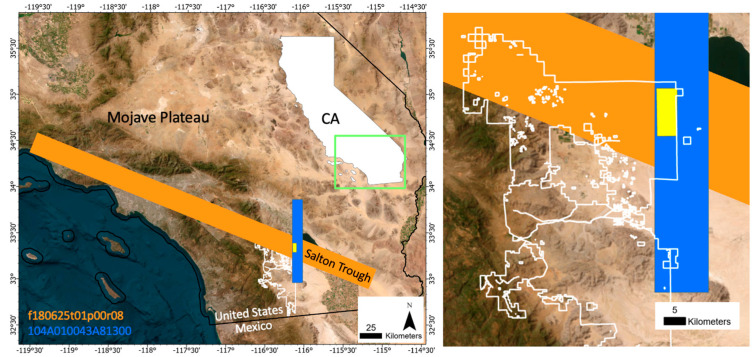
Index map. Anza-Borrego Desert State Park (white outline; both panels) spans regional geologic, topographic, and ecological gradients in California, USA. This study uses one AVIRIS flight line (orange) and one WorldView-3 image (blue). The analysis focused on the triple intersection (yellow rectangle; both panels) of the park boundary with AVIRIS flight line (orange) and WorldView-3 image (blue). True color basemap. The inset map shows the entire state of California; the green box overlay shows the extent of the left panel; and the gray box overlay shows the extent of the right panel.

**Figure 2:**
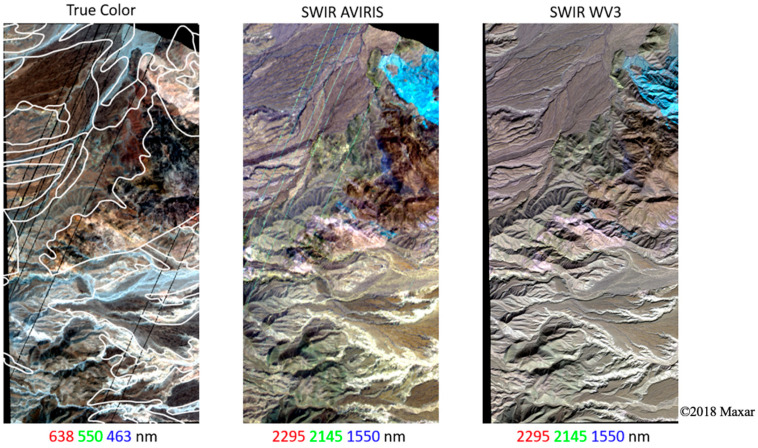
Study area (extent of yellow box in Figure 1). True color AVIRIS image (**left**) with geologic unit boundary vectors overlaid (white; [25]), compared to SWIR false color composites of AVIRIS (**center**) and WorldView-3 (**right**). Compared to the true color imagery, geologic information is more apparent in the SWIR composites, particularly the carbonate absorptions in the marble unit (cyan; NE corner of all images). Note that AVIRIS is spatially coarser than WV3 (15 m versus 4 m) but spectrally finer (10 nm imaging spectroscopy vs. broadband multispectral). Linear 2% stretch applied to each image.

The AVIRIS flight lines were cropped to a subset of the Anza-Borrego park boundary overlapping with WV3 imagery (Figure 1; green rectangle). Bands 1–5, 104–113, and 154–183 (corresponding to 366–405, 1323–1412, and 1821–2087 nm, respectively) were excluded due to atmospheric contamination and/or sensor noise. Some bad lines were apparent upon visual inspection and were excluded from subsequent analysis (Figure 2; NNE-SSW trending lines).

The WV3 data were obtained as digital numbers (DNs) and corrected to exoatmospheric reflectance following the approach of [27]. Additional orthorectification was also performed using ground control points provided with the image data following [28].

### 3.2. Variance-Based Characterization

**Figure 3:**
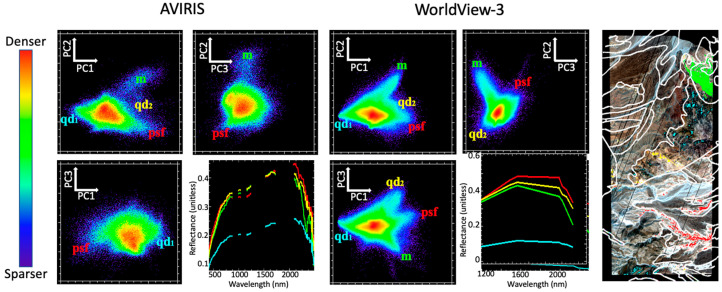
Spectral feature space comparison for AVIRIS vs. WorldView-3 reflectance. PC 1 vs. 2 feature spaces of both sensors share similar topology and geometry. Endmember spectra (lower right for each sensor) correspond to the Palm Springs Formation (psf), marble (m), and quartz diorite (qd_1_) geologic units. The fourth endmember in WV3 corresponds to a different spatial subset of the pixels representing the quartz diorite geologic unit (qd_2_), likely related to intra-unit mineralogical variation within the unit that was not represented by the geologic map.

Figure 3 shows the low-order spectral feature space for both AVIRIS and WV3 reflectance as characterized by Principal Component Analysis (PCA). Three endmember (EM) spectra were manually selected from the apexes of the point cloud. These EMs corresponded to the pink-gray laminated sandstones of the Palm Spring Formation (psf; red), carbonate-rich marble of the Peninsular Range metamorphic complex (m; green), and light gray metamorphosed quartz diorite (qd_1_; cyan). All three endmembers were clearly identifiable in both the AVIRIS and WV3 feature spaces despite the differences in spectral and spatial resolution.

Interestingly, a fourth EM was clearly distinct in the WV3 imagery but not in the AVIRIS data. This EM corresponded to the far SW reach of the variably metamorphosed quartz diorite basement map unit (qd_2_; yellow). This is the same geologic map unit that is captured by the cyan EM. Likely candidates for this reason could be specific mineralogic variations such as percent silica or grain size, bedrock mineralogic and chemical variation related to protolith or metamorphic grade, or surface textural variations related to weathering and erosion, but further fieldwork is needed to confirm this. This is a good example of how both spectral and spatial resolution can be useful to help inform field targeting of new observations during the process of creating a geologic map. The implications for the research questions driving this study are discussed below.

**Figure 4:**
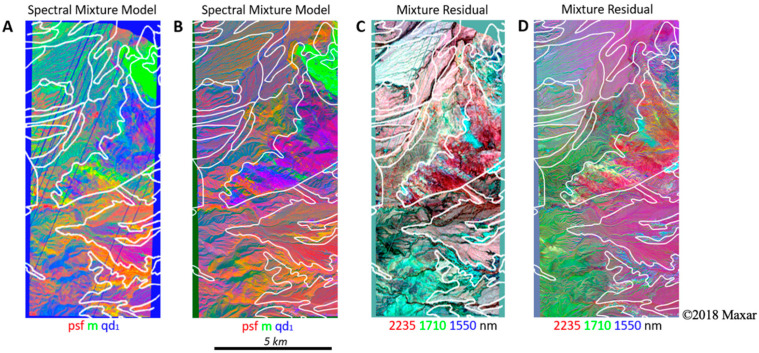
Spectral mixture models and residuals. The results are shown for both AVIRIS (**A**,**C**) and WorldView (**B**,**D**). For spectral mixture models (**A**,**B**), red corresponds to the Palm Spring Formation (psf); blue corresponds to marble (m); and green corresponds to quartz diorite (qd_1_). For the spectral mixture residual (**C**,**D**), the spectral mixture model residual is shown at 2235, 1710, and 1550 nm (red, green, and blue, respectively) wavelengths. Potentially useful geologic information, indicated by the diversity of colors, is clearly present in the residual both within and among geologic units. A linear 2% stretch was applied to each image.

The psf, m, and qd_1_ EMs were then used to perform spectral mixture analysis [29,30,31], with wavelength-explicit retention of the mixture model residual [20]. Endmember fraction images from the AVIRIS and WV3 mixture models (Figure 4A and B, respectively) effectively map areas dominated by each EM lithology. False color composites of AVIRIS and WV3 mixture residuals (Figure 4C and D, respectively) highlight areas with higher wavelength-specific misfit of the model, highlighting differences in the curvature of spectral continuum and narrowband absorption features which correspond to additional mineralogical diversity which is not well fit by the simple 3-EM mixture model.

**Figure 5:**
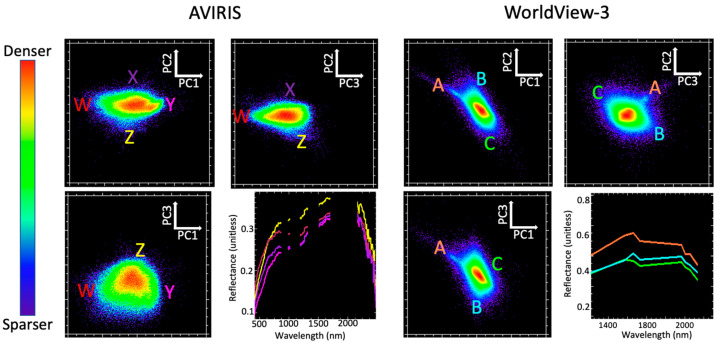
Mixture residual spectral feature space. Regions associated with different geologic features are identifiable for both sensors, but substantially more spectral information can be obtained from AVIRIS than WV3. Specifically, AVIRIS captures differences in VNIR curvature and amplitude, and narrow SWIR absorptions, which are not resolved by WV3. The reflectance spectra for endmembers identified from the MR feature space (lower right for each sensor) correspond to W, X, Y, and Z in the AVIRIS image and A, B, and C in the WorldView-3 image.

The spectral feature space of the mixture residual (MR) (Figure 5) captures additional low-variance spectral variability which is not modeled by the 3 EMs forming the mixture model. For both AVIRIS and WV3, the low-order MR feature space is characterized by EMs with subtle but geologically interesting spectral differences. The AVIRIS spectra demonstrate that MR EMs contain differences in both curvature of the VNIR continuum and specific SWIR absorption features. Spectral information from WV3 is inherently more limited but does include some clear differences in SWIR signals—as well as considerably finer spatial information, manifested as denser, more populated bivariate distributions. The apexes of the MR-based feature space are considerably more poorly defined than for the reflectance-based feature space, but regions proximal in the MR feature space are still observed to cluster geographically in the map space.

### 3.3. Partition of Variance

**Figure 6:**
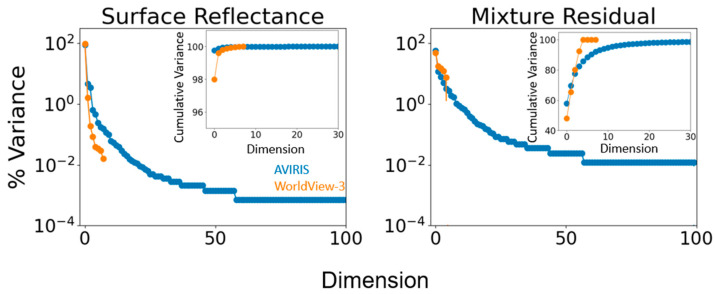
Partition of variance. Normalized eigenvalue distributions are shown for surface reflectance (**left**) versus mixture residual (**right**), as well as for AVIRIS (blue) and WorldView-3 (orange). Inset shows the cumulative variance. AVIRIS reflectance shows greater spectral dimensionality than WorldView-3, with 99% of image variance requiring 7 dimensions for AVIRIS versus only 2 dimensions for WV3. Between-sensor differences in dimensionality are further pronounced in the mixture residual, with AVIRIS capturing 90% variance in 7 bands and 99% variance in 39 bands, but with WorldView-3 capturing 90% variance in 4 bands and 99% variance in 5 bands. WorldView-3 residual eigenvalues are numerically indistinct from zero after the 5th dimension.

Eigenvalue distributions reveal that seven dimensions are required to capture >99% of variance in AVIRIS reflectance, but only two dimensions are required for WV3 (Figure 6, left). Further investigation of variance partitioning for each mixture residual further illustrates the additional spectral information present in AVIRIS: e.g., 5 dimensions capture >99% of WV3 MR variance, while 39 dimensions are required to capture the same fraction of AVIRIS MR variance (Figure 6, right). These results generally agree with previous comparisons of multispectral and spectroscopic sensors in other geologic settings [20].

### 3.4. Topology-Based Characterization 

**Figure 7:**
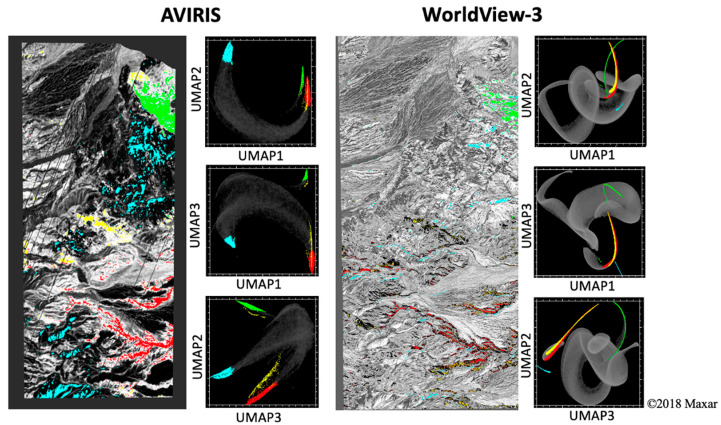
UMAP feature space comparison. For both AVIRIS and WorldView-3 reflectance imagery, nonlinear dimensionality reduction via UMAP complements linear decomposition by PCA, revealing considerably more apparent clusters in regions shown to be diffuse in low-order PC space. EMs follow the same color scheme as previous figures (red: PSF, cyan: qd_1_, green: m, and yellow: qd_2_). Note the enhanced separability of red and yellow clusters in AVIRIS relative to WV3.

Nonlinear dimensionality reduction was performed for both the AVIRIS and WV3 reflectance and residual images using Uniform Manifold Approximation and Projection (UMAP) [32]. The number of nearest neighbors used was 50, and the embedding dimension was 3. For both AVIRIS and WV3 data, UMAP demonstrates enhanced clustering relative to variance-based characterization (Figure 7; compare to Figure 5).

**Figure 8:**
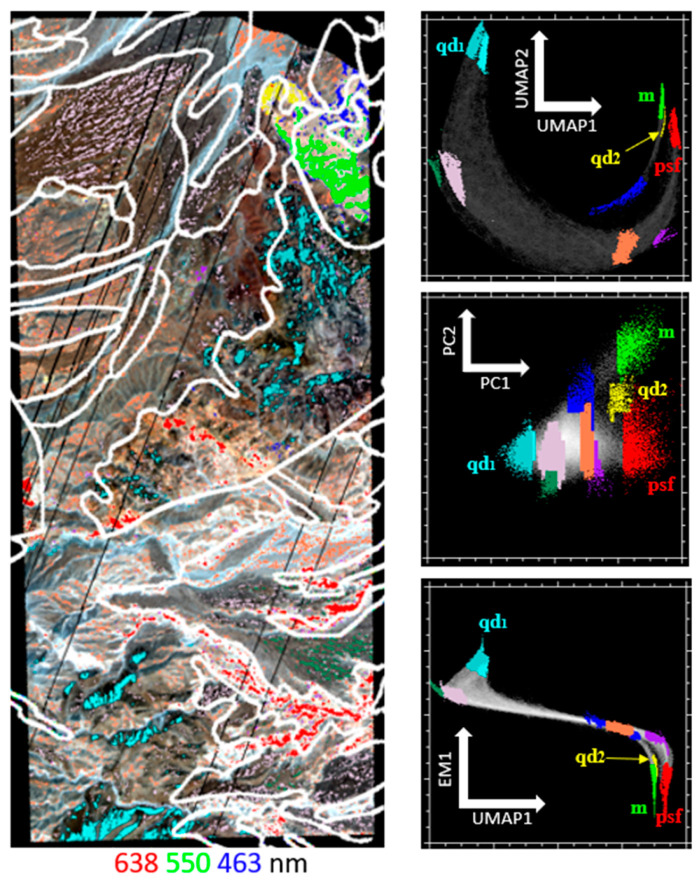
Joint characterization of AVIRIS reflectance. UMAP 1 vs. 2 feature space (**top right**). The clusters were manually selected on the UMAP space and overlaid on PCA space (**middle right**) and joint space (**bottom right**). The clusters identified from UMAP also generally cluster in PC space but are much more diffuse. These clusters also persist in joint space, with the additional benefit of physical interpretability provided by the abundance of one of the spectral mixture model endmembers. The true color AVIRIS image (**left**) with UMAP clusters and geologic unit boundaries overlaid (white) shows the relationship between statistical clustering in UMAP feature space and geographic clustering of geologic features. The EMs are labeled on each scatter plot.

Clusters identified with UMAP correspond to features not readily identifiable from variance-based characterization alone (Figure 8). Clusters identified from the UMAP space (upper right) reveal further clustering within the EMs identified from the PC space, as well as several additional clusters (sea green, magenta, yellow, coral, and purple) that are apparent on UMAP but diffuse or otherwise indistinct in the PC space (middle right). Joint characterization (lower right) combines variance- and manifold-based metrics, effectively partitioning UMAP clusters using physically interpretable variance-based signals.

When the geologic unit boundaries are overlaid on the UMAP image (Figure 8, left), spectral clusters do not always neatly fit into a single geologic unit. This is a graphical representation of the simplifying decisions made during geologic mapping. Local geologic complexity and small-scale variations in bedrock, the surface expression of loose rocks, local accumulations of young sediments, and the spatial complexity of surface processes leads to a real-world earth surface system that is simplified into lithologic units for geologic interpretation. This fundamentally appears more complex when imaged from an aerial or satellite platform than when visualized on a map product. Specific examples of this type of area, and the importance of this complexity for applying this type of data product to geologic interpretation are discussed below.

### 3.5. Spectral Variability within and among Mapped Geologic Units

**Figure 9:**
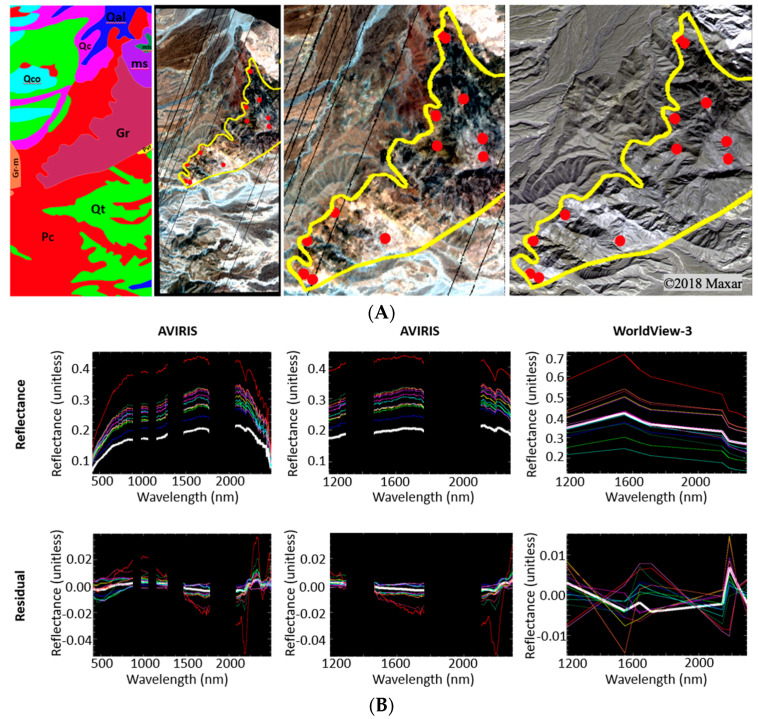
Intra-unit heterogeneity. (**A**) Geologic map units overlaid on the study area (**left**). Granite (Gr) unit highlighted in yellow on the AVIRIS true color image (**center left**). The enlarged view of the Gr unit on AVIRIS (**center right**) and WorldView-3 (**right**) highlights differences in spatial resolution. Single-pixel spectra are marked by red circles. (**B**) The average spectrum of the Gr unit (white line) is compared to the 12 single-pixel spectra selected within the unit for the reflectance (**top row**) and residual (**bottom row**) of both AVIRIS and WorldView-3. The left column row displays the full spectral extent of AVIRIS, while the middle column trims the AVIRIS spectra to the extent of the WorldView-3 column on the right. Differences between the single-pixel reflectance spectra and the Gr unit average spectrum are more clearly identifiable in the residual spectra than in the reflectance spectra and in the AVIRIS spectra than in the WorldView-3 spectra. All reflectance images are shown in true color.

For comparison of information contained within a geologic unit, we examine more detailed spectral data from the plutonic bedrock geologic unit (variably metamorphosed quartz diorite; Figure 9). Pixel spectra from 12 locations within the quartz diorite bedrock unit (yellow vector) were selected to investigate intra-unit variability (A: red dots). For both sensors, subtle differences between mean spectra for the granite unit and individual pixel spectra were accentuated by the mixture residual. Interpretability of geologic information in AVIRIS spectra is clearly greater than in WorldView-3 spectra (B). This reinforces the result from the partition of variance, further suggesting that the spectral differences in information content between AVIRIS and WorldView-3 are amplified by the mixture residual, relative to reflectance. The quartz diorite bedrock lithology includes a nontrivial level of internal heterogeneity that includes characteristic variability such as grain size variation, local mineralogical composition, the grade and pervasiveness of metamorphism, and variations in surface texture. Such factors likely explain the majority of this intra-unit spectral variability.

## 4. Discussion

### 4.1. Research Questions Revisited

#### 4.1.1. Question 1—Spectral Range and Resolution

For multispectral imagery, it has been known for decades that a ternary mixing space can capture 97% or more of the variance in terrestrial landscapes [33,34,35,36,37]. For the dimensionality of imaging spectroscopy data, historical analyses have necessarily been less spatially and temporally comprehensive due to the historical paucity of data [20,38,39,40,41], but have also shown three dimensions to capture roughly 95% or more of variance over large areas. Comparisons have likewise demonstrated similar low-order feature space geometry and topology in datasets spanning a range of spatial and spectral resolutions [20,42]. In at least one study evaluating simulated WV3 data, sufficient information was shown to be present in multispectral SWIR to capture geologically diagnostic information about chemical weathering [3].

Despite these similarities, the intrinsic dimensionality of spectroscopic imagery is consistently demonstrated to be substantially greater than multispectral imagery, with estimates ranging from over 100 dimensions containing unique information [39,40], to the 20s to 40s [43]. The mixture residual retains these higher order dimensions for exploratory analysis [20]. In the present study, the mixture residual eigenvalues clearly demonstrated the much greater geologic information content of AVIRIS relative to WV3, with 39 additional dimensions required to account for >99% of AVIRIS residual spectral variance, compared to only 3 for WV3. This quantitative result was further supported by two qualitative assessments. First, visual comparison of the false color composites of the reflectance (Figure 2) and residual (Figure 4) confirmed clear geologically meaningful variation outside of prominent outcroppings in the residual. Second, comparison of pixel spectra to the average spectrum of an example rock unit (Figure 9) demonstrated that while WorldView-3 detected the existence of a signal in the MR, AVIRIS spectral resolution was required to resolve the shape and curvature of specific absorptions [16,44,45,46,47].

Differences in spatial resolution were also observed to yield important differences in geologic information content. Specifically, WorldView-3 was clearly better able to resolve geologically relevant topographic features such as ridges and peaks than AVIRIS (Figure 2). This difference in spatial resolution can also affect downstream analyses, for instance in cases where subpixel variation and spatial adjacency can impact the accuracy of mineralogical mapping algorithms [48,49]. But despite these visible differences in spatial resolution, the low-order PC-based spectral feature spaces demonstrated important consistencies in topology and endmember character, in agreement with previous analyses [42]. The primary benefit of increased spatial resolution in this study thus came in the form of improvement in visual interpretation. This is not negligible, as higher resolution could help geologists improve strategies for targeting regions of interest for field investigation, identify outcrops at finer spatial scales, and better interpret the outputs of data clustering algorithms. As this approach is designed to complement traditional field mapping, the necessary spatial resolution will fundamentally depend on the accuracy required for the geologic task at hand, whether that be drawing strict boundaries around a geologic unit or trying to gain a general sense of the continuity of geologic outcrop in an area.

Furthermore, we note that the viewing conditions were considerably different between the spaceborne WV3 and airborne AVIRIS. Specifically, the solar position was considerably different between the late June AVIRIS and the late October WV3. The sensor elevations and off-nadir view angles were also different. Vegetation phenology may also be a factor (where present in this sparse desert environment). It is notable that the low-order feature space and spectral endmembers were so consistent across such major differences in sun-sensor geometry, with potentially important bidirectional reflectance distribution function (BRDF) effects. The impact of BRDF effects on dimensionality and feature space characterization of imaging spectroscopy data for geologic mapping are an important and interesting avenue of future work which could potentially be addressed if future data coverage with more favorable acquisition geometry were available.

#### 4.1.2. Question 2—Linear versus Nonlinear Characterization

Linear (PCA-based) characterization of the spectral feature spaces of reflectance (Figure 3) demonstrates similar topology and endmember spectra for the AVIRIS and WV3 datasets. Furthermore, the finer spatial resolution of WV3 allows for an additional (qd2) endmember to be discerned which is present but not clearly identifiable in the low-order feature space of the AVIRIS imagery. For both sensors, the endmembers correlate to known geologic features. 

Additionally, linear characterization of the MR reveals further insight into the sensor intercomparison. Specifically, comparison of the eigenvalues of the reflectance versus residual (Figure 6) quantifies the greater information content of AVIRIS spectra relative to WV3. Qualitative examination of the MR images (Figure 4) also shows greater complexity in AVIRIS. 

Nonlinear characterization of both reflectance and MR image data provides yet further information. The UMAP feature spaces were found to both capture the endmembers of the reflectance PCA (Figure 7) and delineate finer clusters within these endmember regions (Figure 8). In joint characterization, using statistical (clustering) information together with physical (mineral EM fraction) signal leveraged the strengths of both approaches and provided a useful combination of both clustering and physical interpretability (Figure 8, bottom right).

#### 4.1.3. Question 3—Implications for Geologic Mapping

Geologic maps are visual representations of conceptual models that explain the observations and relationships amongst different lithologies and geologic structures. As such, the level of information used by a mapper to (1) develop a model, (2) translate it onto a map, and (3) iteratively test its implications and predictions are inherently of high dimensionality. In fact, much of the “boots on the ground” time of a geologic mapper involves an individual tracking down field data that could provide critical evidence to disprove or support a hypothesis. In this regard, the introduction of a new type of high dimensional IS-based information is inherently useful for integrating into the workflow of an evolving geologic interpretation. Approaches like JC and MR increase the information content of IS data accessible to geologists, enabling increased adoption for geologic mapping work. The higher dimensionality of IS data is welcomed by geologists, as it enables a fundamentally better reflection of real-world complexity than was possible with earlier remote sensing products.

### 4.2. Limitations

We emphasize that, for geologic mapping, remote sensing is primarily a tool to be used in conjunction with field studies. Advances in IS sensor technology, combined with approaches such as JC, can cluster data in statistically and geologically meaningful ways, in both pre- and post-field work. Nevertheless, field validation of mineralogical and lithological signals is still crucial to ensuring accuracy, especially in areas with coarse spatial and/or spectral resolution, where subpixel (linear and nonlinear) mixing and spectral non-uniqueness could affect analyses. Furthermore, in the construction of the spectral mixture model, as well as in the determination of informative clusters for JC, endmembers were manually selected, and region interpretation was confirmed by visualization relative to both the imagery and geologic map. There thus remains a burden on the researcher to think critically in determining meaningful bounds in the statistical space and to use the information from image-derived spectra to develop accurate geologic interpretations in the context of other types of data.

### 4.3. Future Work

#### 4.3.1. Integration with Other Algorithms

An exciting avenue for future work could be the integration of the JC and MR algorithms with algorithms for pixelwise mineralogical mapping such as Tetracorder [16]. Utilizing thousands of mineralogical spectra characterized by the USGS Spectroscopy Lab [50], this approach characterizes the existence and abundance of dominant modes of surface mineralogy [16] and has seen extensive use in the analysis of airborne IS data [51,52,53]. It is possible that the low-variance and topological information captured by MR and JC could provide a useful complement to the strengths of such mineral mapping algorithms.

#### 4.3.2. Data Fusion

JC could also be applied to additional remote sensing modalities. In particular, thermal infrared (TIR) emissivity has also been shown to contain useful geologic information, particularly for carbonates, silicates, sulfates, and clays [54,55]. Furthermore, analysis of the intrinsic dimensionality of TIR images from the HyTES sensor found comparable dimensionality to overlapping AVIRIS classic imagery, suggesting that this wavelength range could provide new and complementary geologic information to VSWIR imagery [41]. While dimensionality calculations can differ based on the technique used, two methods agreed in finding the fusion of AVIRIS and HyTES imagery to increase dimensionality relative to either sensor alone [41].

The information provided by active remote sensing modalities like LiDAR and SAR also offer avenues for future work. Efforts to combine spectroscopic and LiDAR data have found that joint approaches can be more effective than either alone, especially in urban settings featuring both spectrally similar features at different elevations (e.g., tall trees versus low-lying shrub canopies) and spectrally distinct features at similar elevations (e.g., grass versus sidewalk) [56,57,58,59]. Such joint datasets could easily be extended to geologic mapping—for instance, where topographic gradients and mineralogical composition could be used together to characterize processes like weathering and erosion.

## 5. Conclusions

Using a subset of Anza-Borrego Desert State Park as a study area, we examine the effect of spatial and spectral resolution on the union of recent analytic advances in the spectral mixture residual and joint characterization. Analysis of the spectral mixture residual reveals that geologically useful information is contained in the AVIRIS mixture model residual which is not present in WorldView-3. Joint characterization using principal component analysis (PCA) and uniform manifold approximation and projection (UMAP) is able to identify geologically relevant spectral clusters for both AVIRIS and WorldView-3. Furthermore, some clusters found by joint characterization transcend previously drawn geologic unit boundaries, providing additional useful information and context outside of established geologic mapping techniques. The utility and salience of this approach for field geologists is likely to grow in the coming years, as the spatial and spectral precision of open Earth datasets continue to advance.

## Data Availability

The AVIRIS data supporting this manuscript can be downloaded free of charge from the web portal listed in the main text. WorldView-3 data were made available through the NASA Commercial Smallsat Data Acquisition (CSDA) program.

## Appendix A. Image Metadata

### Appendix A.1. AVIRIS Flight Line ID

f180625t01p00r08

### Appendix A.2. WorldView-3 Scene ID

Image ID: 104A010043A81300

Image Clouds: 0.0%

Image Off Nadir: 24.7°

Bands: SWIR-BANDS

Sun Elevation: 43.0°

Max Target Azimuth: 348.6°
